# Impact of High Aspect Ratios and Reinforcing Indexes on Mechanical Properties of Hybrid and Non-Hybrid Chopped Glass Fiber Reinforced Concrete

**DOI:** 10.1021/acsomega.2c05878

**Published:** 2022-12-07

**Authors:** Ferit Cakir, Pinar Yildirim, Beste Kocak Dinc, Mazem Balaban

**Affiliations:** † Department of Civil Engineering, Gebze Technical University, Cayirova Campus, Gebze, Kocaeli41400, Turkey; ‡ Department of Civil Engineering, Istanbul Aydin University, Halit Aydin Campus, Kucukcekmece, Istanbul34295, Turkey

## Abstract

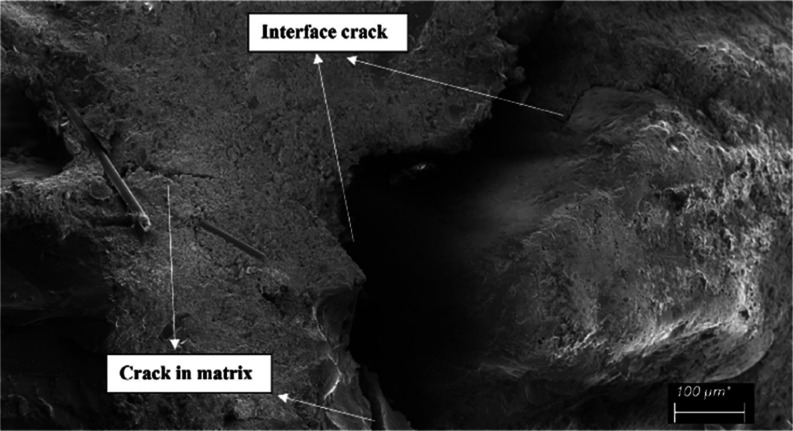

Due to technological advancements, concrete can be currently produced with varying strengths and durability based on its intended use. However, in many applications, concrete still needs to be improved in terms of its mechanical and physical properties. The addition of fibers to concrete is one of the most widely used methods for improving its mechanical and physical properties. The study focuses on the effects of the high aspect ratios and reinforcing indexes on the mechanical properties of the hybrid and non-hybrid chopped glass fiber reinforced concrete (CGFRC). In this study, the glass chopped fibers (GCFs) (fiber diameter, ϕ = 0.015 mm) with four different volume fractions (0, 0.5, 0.75, and 1%) and four different lengths (3, 6, 12, and 24 mm) were mixed into the concrete considering the aspect ratios between 200 and 2800 and the reinforcing indexes between 1 and 42. A total of 51 samples were prepared for the study that included 3 control, 36 non-hybrid, and 12 hybrid samples. Then, the flexural strength and compressive strength tests were conducted on the CGFRC samples. To obtain detailed information about fiber pullout, fiber breakage, debonding, or cracking in the matrix, digital microscopy and scanning electron microscopy examinations were performed. The flexural strength of the hybrid samples increased with the higher aspect ratios and reinforcing index values, whereas the flexural strength of non-hybrid samples decreased with the higher aspect ratios and reinforcing index values in the CGFRCs. Moreover, all non-hybrid and hybrid CGFRC samples had lower compressive strengths than the control samples in terms of compressive strength. With an increase in the fiber volume fraction, the mixing and workability of the samples considerably decrease, and the increase of the fiber volume fraction caused brittle fractures in concretes to be transformed into ductile fractures.

## 1 Introduction

Despite its high compressive strength, concrete is a brittle material with low tensile strength. Therefore, cracks and possible failures of concrete structures occur when tensile stress exceeds the corresponding low strength.^1^ Currently, concrete materials are typically reinforced with steel rebars. Because steel rebars are heavy construction materials, their mass increases the forces on the structures. Hence, researchers have recently been experimenting and developing new strengthening materials for concrete structures by using different engineering materials such as chopped steel fibers, fiber-reinforced polymers, steel plates, and ferrocement.^2−5^ Today, adding additives to concrete or reinforcing concrete with different materials is one of the most preferred methods to improve the mechanical properties of concrete due to its significant advantages such as easy application and minimum change in the overall size. Especially, improving the mechanical and physical properties of concrete by adding chopped fiber to concrete has been a method that has been applied for many years. While the use of fiber in concrete was made with goat hair, horse mane, straw, and even human hair in the past, the use of chopped fibers such as steel, carbon, glass, aramid, or basalt has currently become widespread.^6−10^ The improvement of concrete performance by using fibers depends on many factors such as fiber type and size, aspect ratio, reinforcing index, and fiber–matrix interaction.^10,11^ Besides, the compatibility of fibers with components of the concrete is very significant for the structural performance of the concrete.

Chopped glass fiber (CGF) is one of the most preferred chopped fibers today. In comparison to other chopped fibers, the CGF has gained substantial attention from the engineering community as a result of its low cost, high tensile strength, high chemical resistance, and insulating properties for large and small concrete structures alike.^6,8−10,12^ Therefore, most previous studies have concentrated on the benefits of chopped fibers on the mechanical performance of concrete. Yuan and Jia^13^ conducted some experiments to study the effect of polypropylene fibers and glass fibers on the mechanical and microstructural properties of the concretes. To prepare the concrete samples, different ratios of water/binder (0.30 and 0.35) and different fiber contents (0.45, 0.90, and 1.35% by volume) were used. The study concluded that the water/binder ratio affected the glass fiber ratio. Furthermore, the glass fibers were more effective at absorbing water in concrete than polypropylene fibers. Cakir^14^ investigated the effects of two types of chopped fibers which were CGFs and chopped basalt fibers on the physical and mechanical properties of polymer mortars (PMs). In the study, three different mixtures were prepared and mechanical tests were conducted on the samples based on different curing times: 7, 14, 21, and 28 days. According to the experimental studies and evaluations, chopped fibers influence the mechanical properties and failure modes of PMs, and CBFs are better additives than the CGFs. Moghadam and Izadifard^15^ studied the effects of steel and glass fiber addition on the behavior of normal concrete at high temperatures. In the study, the fiber volume fraction was 0.25% and experimental temperatures ranged from 28 to 800 °C. At high temperatures, steel fibers improved compressive, tensile, and shear strengths in a range of 9–27, 8–198, and 1–22%, respectively. In specimens containing the glass fiber, the compression and tensile strength were both improved by 1–18 and 19–213%, respectively. Ganta et al.^16^ conducted a study to determine how fiber type and aggregate content affect the hardened and durability properties of self-consolidating concrete. In the present experimental program, steel, glass, and steel/glass hybrid fibers were tested. Based on the results, the hybrid reinforced self-consolidating concrete shows good mechanical and durability performance compared to other mixtures. Ali and Qureshi^17^ examined the effects of glass fibers on concrete with recycled coarse aggregates (RCAs) concerning mechanical performance and durability. The results of testing show that 50% RCA concrete outperforms the plain natural coarse aggregate (NCA) concrete at 0.5% GF in overall mechanical performance (compressive, split tensile, and flexural strength). Test results indicated the 50% RCA concrete outperforms the NCA at 0.5% GF in terms of overall mechanical strengths which were compressive, split tensile, and flexural strength. Riad et al.^18^ assessed the behavior of reinforced concrete beams under different fire and cooling conditions by adding discrete glass fibers. In this study, a series of 18 concrete beams with different compressive strengths were tested to analyze the behavior of RC beams containing discrete glass fibers under various cooling and fire conditions. Finally, the study recommended that the percentage of discrete glass fibers in concrete should not exceed 0.5%. Kizilkanat et al.^19^ studied the effect of basalt and glass fibers on high-strength concrete. Several experiments were conducted using concrete samples produced with different fiber volumes (0.25, 0.50, 0.75, and 1%). The fibers reduced the workability of the concrete but did not have any significant effect on its modulus of elasticity. The compressive strength of the samples also increased slightly with fiber additions. In addition, the study pointed out that fiber additions enhanced flexural strength. Tassew and Lubel^12^ investigated the effects of the CGFs on the mechanical and rheological properties of the ceramic concrete binder with phosphate cement. It was shown that by increasing fiber content in the mortar mixture, hardness, bending, and shear strength increase, but compressive strength and elasticity are not affected. In addition, the fiber reinforcement decreased the workability of the mortar mixture.

Based on the previous literature, it is clear that many studies have been conducted on the use of fibers in concrete to enhance its physical and mechanical properties. However, the studies conducted are still insufficient and there are still unresolved or inconclusive issues concerning the improvement of fiber materials for concrete. Therefore, the study focuses on the effect of the high aspect ratios and reinforcing indexes on the flexural and compressive strengths of the CGF reinforced concrete (CGFRC). For this purpose, 51 CGFRC samples, having 3 control, 36 non-hybrid, and 12 hybrid samples, were constructed, and then, three-point bending strength and uniaxial compressive strength tests were conducted on the samples. In this study, the GCFs (fiber diameter, ϕ = 0.015 mm) with four different volume fractions (*V*_f_ = 0, 0.5, 0.75, and 1%) and four different lengths (*L*_f_ = 3, 6, 12, and 24 mm) were mixed into the concrete considering the aspect ratios (*L*_f_/ϕ) between 200 and 2800 and the reinforcing indexes between 1 and 42.

## 2 Materials and Methods

### 2.1 Materials

In this study, the CGFRCs were produced by using silica aggregate, cement, and CGFs. For the samples to be compatible with each other and not to mislead the experimental results, all materials used in the study were used from the same material packages.

#### 2.1.1 Silica Aggregates

Because silica sand has a high hardness value, resistance to abrasion and weather conditions, and chemical stability,^20^ it was used as an aggregate in the samples. The chemical properties of the aggregates according to their particle size are shown in Table 1. Furthermore, the silica sand used in this study was sieved according to the TS 706 EN 12620 Standard (Aggregates for Concrete).^21^ In addition, the water absorption tests and specific gravity tests were carried out following TS EN 1097-6 Standard^22^ (Tests for mechanical and physical properties of aggregates—Part 6: Determination of particle density and water absorption). The results of the experiments are presented in Table 2.

**Table 1:** Chemical Properties of the Aggregate

	aggregate sizes			
chemical composition	0.3–1 mm	1–2 mm	2–3 mm	3–5 mm
MgO	0.10	0.06	0.06	0.06
Al_2_O_3_	0.245	1.86	1.86	1.86
SiO_2_	98.86	94.15	94.15	94.15
CaO	0.01	0.39	0.39	0.39
Fe_2_O_3_	0.148	0.46	0.46	0.46
SO_3_		0.10	0.10	0.10
K_2_O	0.03	1.56	1.56	1.56
Na_2_O	0.02	1.12	1.12	1.12

**Table 2:** Experimental Properties of the Aggregate

aggregate type	relative density (mg/m^3^)	water absorption (%)	water content (%)
silica sands	2.62	2.24	0.04

#### 2.1.2 Cement

For the preparation of the samples, Portland cement was used as a binder material. The cement used in this study was CEM I/42.5R cement from Akçansa Cement Company. The chemical and physical characteristics of the cement are presented in Table 3.

**Table 3:** Chemical and Physical Properties of the Cement (Obtained From Akçansa Cement)

Physical Properties	
color	gray
specific gravity (mg/m^3^)	3.11
Fineness	
specific surface—blaine (cm^2^/gr)	3810
residue on 45 μm sieve (%)	3.1
residue on 90 μm sieve (%)	0.2
soundness (Le Chatelier) (mm)	1.0
Mineralogical Composition	
C_3_S	63.91
C_2_S	5.48
C_3_A	7.39
C_4_AF	10.07
Setting Time (Vicat Test)	
initial (min.)	137
finish (min.)	216
Mechanical Properties	
early strength-2 day (MPa)	39.0
standard strength-28 day (MPa)	61.9

#### 2.1.3 Chopped Glass Fibers

The glass fiber produced by Dost Kimya was used as the chopped fiber to focus on the effect of the CGFs on the mechanical properties of concrete samples. In the study, the fiber length (*L*_f_) was preferred as 3, 6, 12, and 24 mm. Technical properties of the CGFs are given in Table 4.

**Table 4:** Technical Specifications of the CGF

fiber type	chopped glass fiber
tensile strength (MPa)	3400
elasticity modulus (GPa)	77
application temperature (°C)	(−60)–(+650)
density (gr/cm^3^)	2.6
fiber diameter (ϕ) (μm)	15
fiber lengths (*L*_f_) (mm)	3, 6, 12, 24

### 2.2 Reinforcing Index

Several factors influence the structural behavior of the CGFRCs, including the geometrical and mechanical properties of the CGFs, aspect ratios (*L*_f_/ϕ), fiber volume fractions (*V*_f_), and reinforcing indexes (RIs). The RI is considered as a key factor when determining how fiber content and aspect ratio affect composite properties.^23−25^ The CECS38^26^ defines a RI as the characteristic value of fiber, as follows
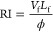
1where, *V*_f_, *L*_f_, and ϕ represent the volume fraction of fiber, fiber length, and fiber diameter, respectively. The use of eq 1 only uses for single fiber types and lengths and it is not possible to use it for hybrid fibers such as different fiber types of different fiber lengths because hybrid fibers have different fiber characteristics. Hence, Almusallam et al.^23^ and Cao et al.^24^ reported a new formula for reinforcing index which is as follows.
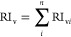
2
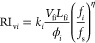
3

In this equation, RI_v_ represents the reinforcing index of hybrid fibers. The suffix *i* indicates the type of fiber. In this equation, *i* can be represented as 1,2,3, and 4, where 1 symbolizes CGF 3 mm, 2 symbolizes CGF 6 mm, 3 symbolizes CGF 12 mm, and 4 symbolizes CGF 24 mm. In fibers, *k*_*i*_ represents the mechanical anchoring coefficient related to the surface shape. In this paper, the surface shape of CGF was considered smooth and straight and assumed weak bonding with the matrix. In this study, the values of *k*_*i*_ were taken as 0.1, 0.2, 0.4, and 0.8 for 3, 6, 12, and 24 mm CGFs, respectively. In eq 3, *f*_*i*_ and *f*_s_ represent the tensile strength of different fiber types and steel fiber, respectively. The index η in eq 3 represents the parameter that is related to fiber type, and for the CGF fiber, it was taken as 1. The calculated RIs are summarized in Table 5.

**Table 5:** Reinforcing Indexes

samples	mixtures	fiber length (*L*_f_) (mm)	volume friction (*V*_f_) (%)	aspect ratios (*L*_f_/ϕ)	reinforcing indexes (RI_v_)
**control sample**	mixture-1		0	0	0
**non-hybridsamples**	mixture-2	3	0.5	200	1
	mixture-3	3	0.75	200	1.5
	mixture-4	3	1	200	2
	mixture-5	6	0.5	400	2
	mixture-6	6	0.75	400	3
	mixture-7	6	1	400	4
	mixture-8	12	0.5	800	4
	mixture-9	12	0.75	800	6
	mixture-10	12	1	800	8
	mixture-11	24	0.5	1600	8
	mixture-12	24	0.75	1600	12
	mixture-13	24	1	1600	16
**hybrid samples**	mixture-14	3, 6, 12	0.25	1400	10.5
			0.25		
			0.25		
	mixture-15	6, 12, 24	0.25	2200	42
			0.25		
			0.25		
	mixture-16	3, 6, 24	0.25	2600	34.5
			0.25		
			0.25		
	mixture-17	3, 12, 24	0.25	2800	40.5
			0.25		
			0.25		

### 2.3 Preparation of the Samples

**Figure 1:**
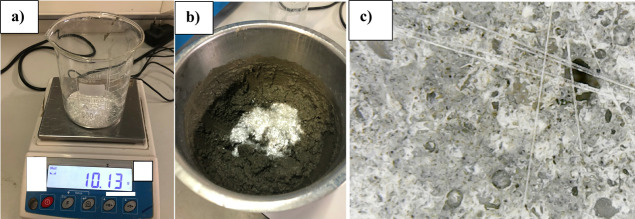
Concrete mixture: (a) weighing the CGFs and (b) addition of the CGFs. (c) Homogeneous distribution of the fibers in the concrete obtained from the digital microscope.

For the experimental studies, the amount of cement and sand added to each mixture was calculated and these materials were individually packaged to maintain the moisture content. The samples were prepared by following the mixture principles specified in TS EN 196-1 (Methods of testing cement-Part 1: Determination of strength).^26^ According to this standard, the Water/Cement (W/C) ratio was preferred as 0.50% in all samples. The GCFs (fiber diameter, ϕ = 0.015 mm) with four different volume fractions (*V*_f_ = 0, 0.5, 0.75, and 1%) and four different lengths (*L*_f_ = 3, 6, 12, and 24 mm) were mixed into concrete with aspect ratios (*L*_f_/ϕ) of 200, 400, 800, and 1600. According to previous studies, the use of 0.25% glass fiber did not have a significant effect on the bending and compressive strengths^19^ and the bending and compressive strengths decreased when the glass fiber content exceeded 1.00%.^27^ In this study, a range of values that offer better results has been selected to assess the mechanical behavior of the fiber producing the additive. To achieve uniform distribution and prevent damage from over-mixing, the components were mixed by a mechanical stirrer for 90 s, with 60 s at low speed and 30 s at high speed (Figure 1).

**Figure 2:**
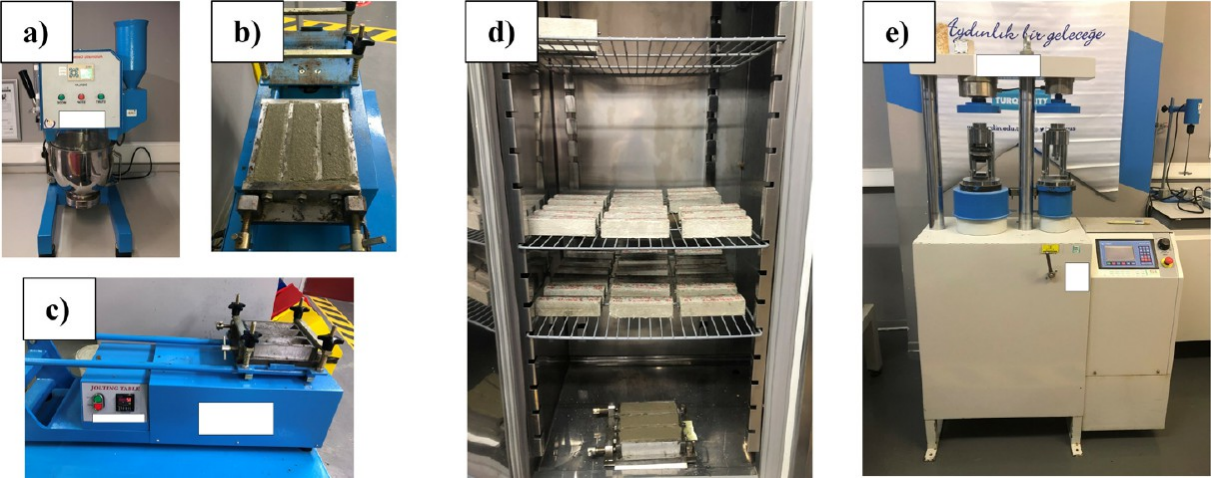
Devices and methods used in the experimental study: (a) automatic mixer, (b) sample molds, (c) shaking table, (d) curing cabinet, and (e) compression and flexural test device.

**Figure 3:**
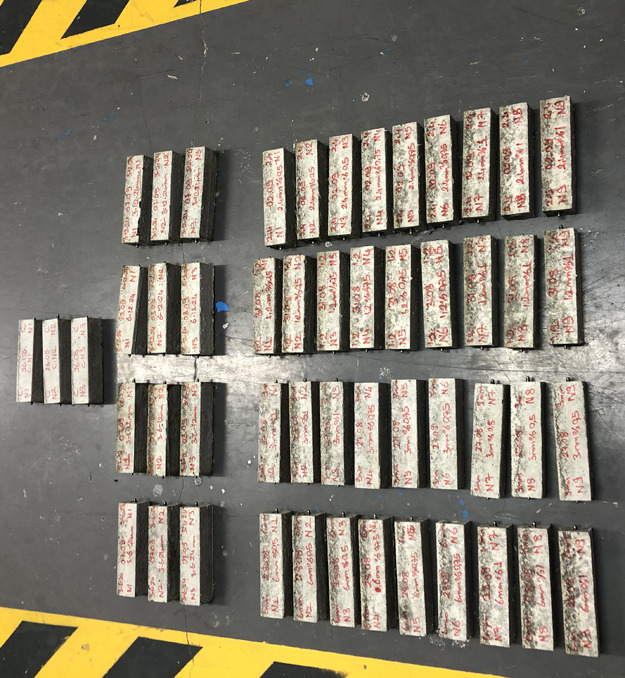
Test samples.

For the flexural and compression tests, the fresh GCFRC mixtures were placed into the 40 × 40 × 160 mm prism molds. Each mold was then vibrated on a shaking table in order to compact the samples. The prepared samples were placed in a curing cabinet for 24 h in molds. After removal from the molds, the samples were placed in a curing cabinet at 20 °C and 90% relative humidity for 28 days. A total of 17 sets (51 pieces) of 40 × 40 × 160 mm sized rectangular prism samples were prepared for the experimental studies. Figures 2 and 3 illustrate the production details, preparation devices, and samples; Table 6 gives the production details.

**Table 6:** Mixing Ratios of Samples

mixtures	amount of fibers gr/(*V*_f_)		
mixture-1	0.00/(0%)		
mixture-2	10.13/(0.5%)		
mixture-3	15.19/(0.75%)		
mixture-4	20.25/(1%)		
mixture-5	10.13/(0.5%)		
mixture-6	15.19/(0.75%)		
mixture-7	20.25/(1%)		
mixture-8	10.13/(0.5%)		
mixture-9	15.19/(0.75%)		
mixture-10	20.25/(1%)		
mixture-11	10.13/(0.5%)		
mixture-12	15.19/(0.75%)		
mixture-13	20.25/(1%)		
mixture-14	5.06/(0.25%)	5.06/(0.25%)	5.06/(0.25%)
mixture-15	5.06/(0.25%)	5.06/(0.25%)	5.06/(0.25%)
mixture-16	5.06/(0.25%)	5.06/(0.25%)	5.06/(0.25%)
mixture-17	5.06/(0.25%)	5.06/(0.25%)	5.06/(0.25%)

## 3 Experimental Studies

**Figure 4:**
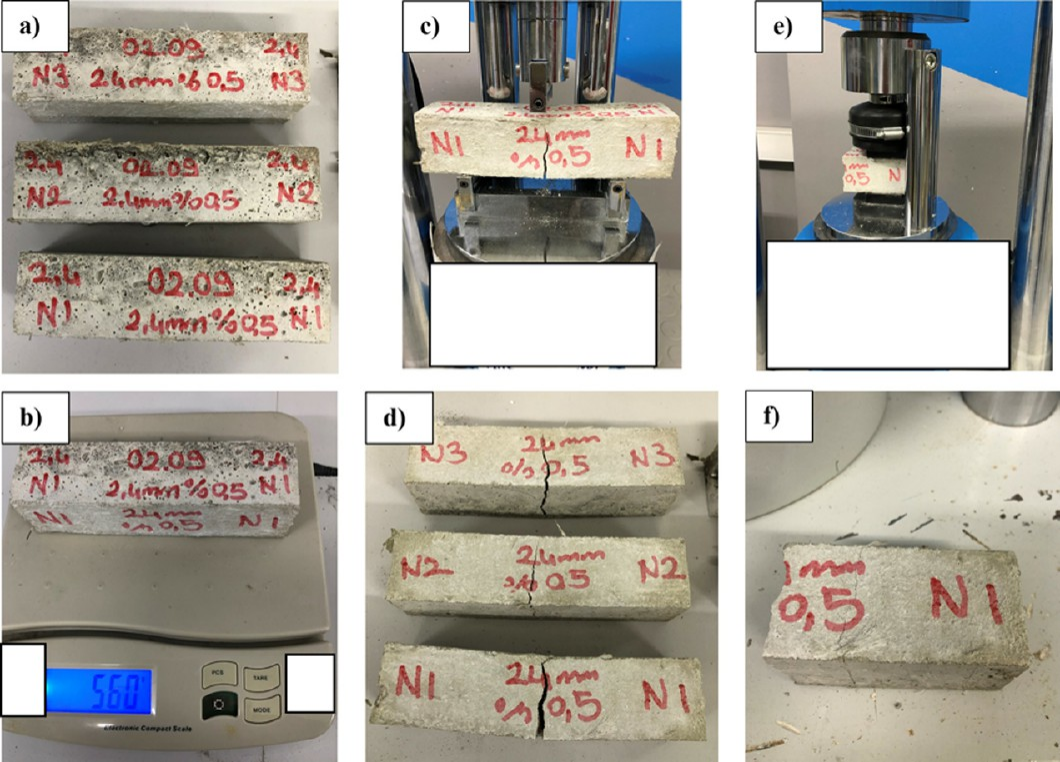
Experimental studies pattern, (a) samples, (b) weighing of samples, (c) three-point bending test, (d) failure patterns after the three-point bending test, (e) compressive strength test, and (f) failure patterns after compressive strength test.

The experimental studies began after the cure period of 28 days. To determine the effect of GCFs on the mechanical properties of the concrete, three-point flexural strength tests and uniaxial compression strength tests were performed. The mechanical tests were conducted according to TS EN 196-1 (Methods of testing cement-Part 1: Determination of strength).^25^ The experimental studies started with determining the densities of each sample, followed by three-point bending tests. According to the Test Procedure (Item 9) section of TS EN 196-1,^25^ each part of the sample divided into two parts was subjected to uniaxial compressive strength tests to determine the compressive strength of each part (Figure 4). All experiments were conducted at the Civil Engineering Laboratories of Istanbul Aydin University (IAU).

## 4 Visual Inspections

Scanning electron microscopy (SEM) and digital microscopy (DM) examinations of the samples were conducted to better understand the fracture process, failure mode, bonding mechanism, and debonding or crack morphology. 1600× DM (Bushman 1600×) and Sigma 300 SEM (Zeiss) were used for visual inspections. Visual inspections were conducted on the specimens that failed the mechanical testing by cutting them through the cross-section of the failed surface. During SEM inspection, samples were coated with gold to prevent electrons from charging them. To obtain the desired magnification, a 5 kV accelerating voltage was applied.

## 5 Results and Discussion

This section focused on understanding the effects of the CGFs on the mechanical properties of concrete and the results of the experimental studies were compared and discussed. As a first step, all data from the experimental studies were combined in Table 6 and normalized to make comparisons meaningful. As can be seen in Table 7, the weight per unit of volume (WPUV) values vary between 2.17 and 2.26 gr/cm^3^. It was determined that WPUV values showed a general decreasing trend with the addition of fiber but did not show a significant change (Table 7). These results are in line with previous literature. According to Cakir,^14^ the addition of glass and basalt fibers to polymer concrete causes modest declines in concrete density. This situation resembles the study of Ates and Aztekin.^28^ It was found that the chopped fibers reduced the density of polymer concrete after fiber addition. According to Seker et al.,^29^ the addition of chopped fibers reduces density in lime-based mortar. Considering the flexural strengths of the samples, it was found that the highest average flexural strengths were obtained as 8.58 MPa in mixture-5. The result showed that this value was almost 9% higher than the control sample. On the other hand, the lowest average flexural strengths were obtained as 6.99 MPa in mixture-4. The result showed that this value was almost 10% lower than the control sample (Table 7). According to obtained results, there was generally a decrease in the flexural strength of fiber reinforcement samples at the same aspect ratio as the volume fraction increased. As reported in the previous literature, Choi and Yuan^30^ and Kizilkanat et al.^19^ reported positive impacts of chopped fibers on the mechanical properties of concrete. Similarly, Cakir^14^ and Shokrieh et al.^31^ emphasize that chopped fibers added to polymer concrete increase their flexural strength.

**Table 7:** Summary of the Experimental Results

mixtures	aspect ratios (*L*_f_/ϕ)	reinforcing indexes (RI_v_)	average weight per unit of volume (gr/cm^3^)	average flexural strength (MPa)	average compressive strength (MPa)	normalized flexural strength	normalized compressive strength
mixture-1	0	0	2.26	7.88	47.97	1.000	1.000
mixture-2	200	1	2.20	7.79	45.82	0.989	0.955
mixture-3	200	1.5	2.19	7.63	43.65	0.968	0.910
mixture-4	200	2	2.26	6.99	43.01	0.887	0.897
mixture-5	400	2	2.23	8.58	46.07	1.089	0.960
mixture-6	400	3	2.23	7.86	44.54	0.997	0.928
mixture-7	400	4	2.21	7.20	44.33	0.914	0.924
mixture-8	800	4	2.26	8.45	46.65	1.072	0.972
mixture-9	800	6	2.22	8.14	42.73	1.033	0.891
mixture-10	800	8	2.17	7.60	41.08	0.964	0.856
mixture-11	1600	8	2.22	7.66	45.03	0.972	0.939
mixture-12	1600	12	2.20	7.46	43.49	0.947	0.907
mixture-13	1600	16	2.19	7.34	41.59	0.931	0.867
mixture-14	1400	10.5	2.20	8.51	40.96	1.080	0.854
mixture-15	2200	42	2.20	8.52	44.76	1.081	0.933
mixture-16	2600	34.5	2.21	8.09	46.64	1.027	0.972
mixture-17	2800	40.5	2.20	7.86	45.22	0.997	0.943

**Figure 5:**
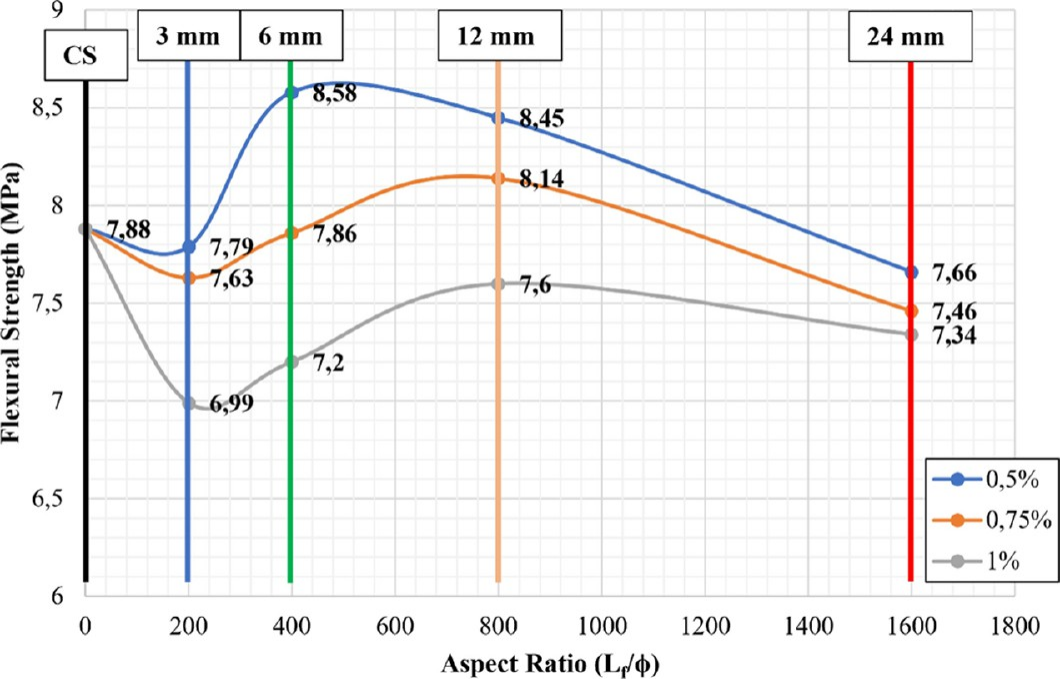
Relationship between flexural strength and aspect ratios for non-hybrid samples.

**Figure 6:**
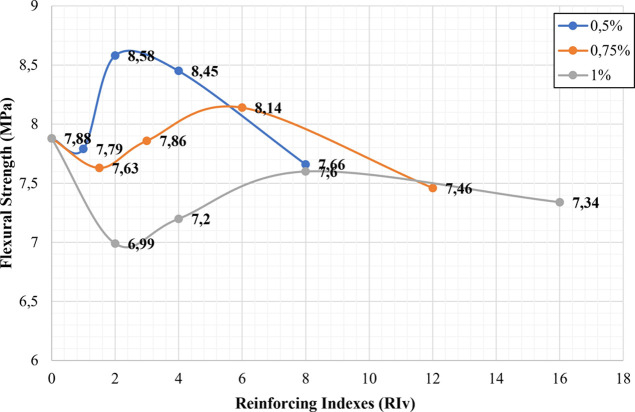
Relationship between flexural strength and reinforcing indexes for non-hybrid samples.

Within the scope of the study, the relationship between the flexural and compressive strengths was also examined considering the aspect ratios and reinforcing indexes. For this purpose, the average values for the flexural and compressive strengths of the samples were compared to each other considering non-hybrid and hybrid samples. As can be seen from Figure 5, the best tensile strength values of the hybrid samples were obtained in the case of 0.5% mixing of 6 mm CGFs with the aspect ratio of 400 and reinforcing index of 2, which was mixture-5 (Figures 5 and 6).

**Figure 7:**
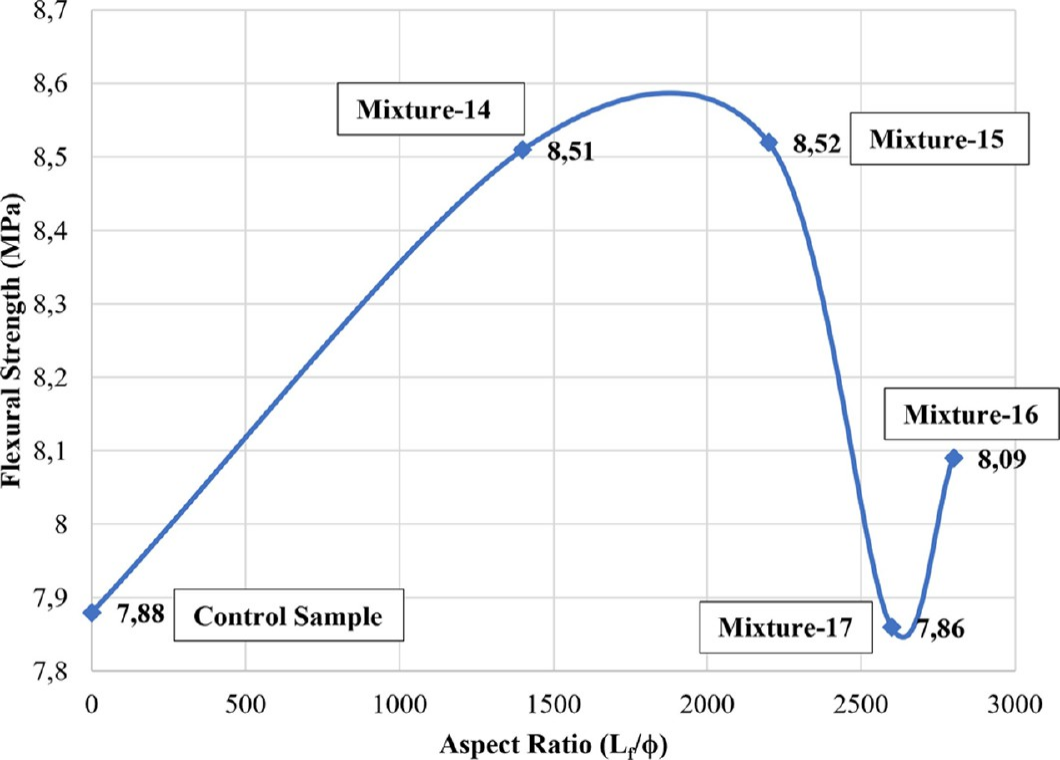
Relationship between flexural strength and aspect ratios for hybrid samples.

**Figure 8:**
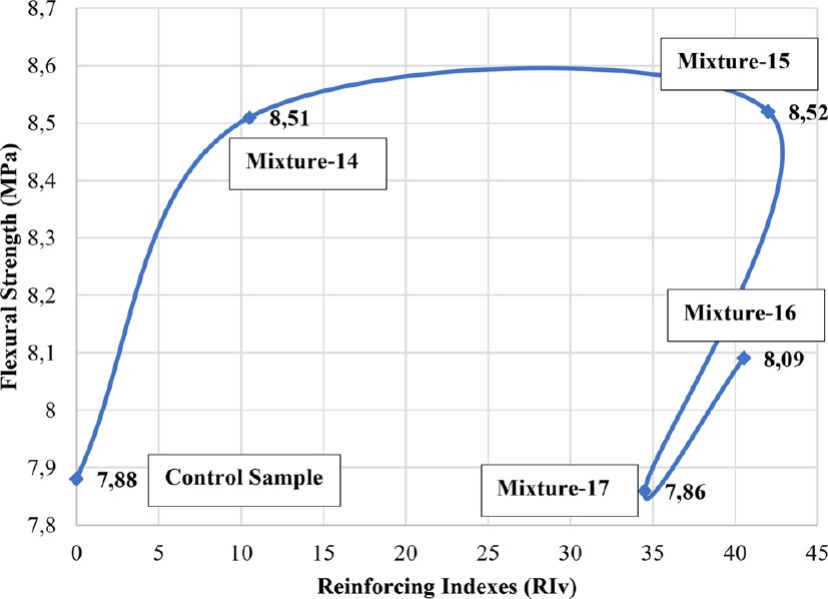
Relationship between flexural strength and aspect ratios for hybrid samples.

When the hybrid samples were examined, it was determined that the sample with the best flexural strength was the mixture-15 sample with an aspect ratio of 2200 and a reinforcing index of 42. According to the data obtained, the flexural strength of the samples with a high aspect ratio and reinforcing index values caused higher increases in hybrid samples (Figures 7 and 8), while this situation was not observed in non-hybrid samples.

**Figure 9:**
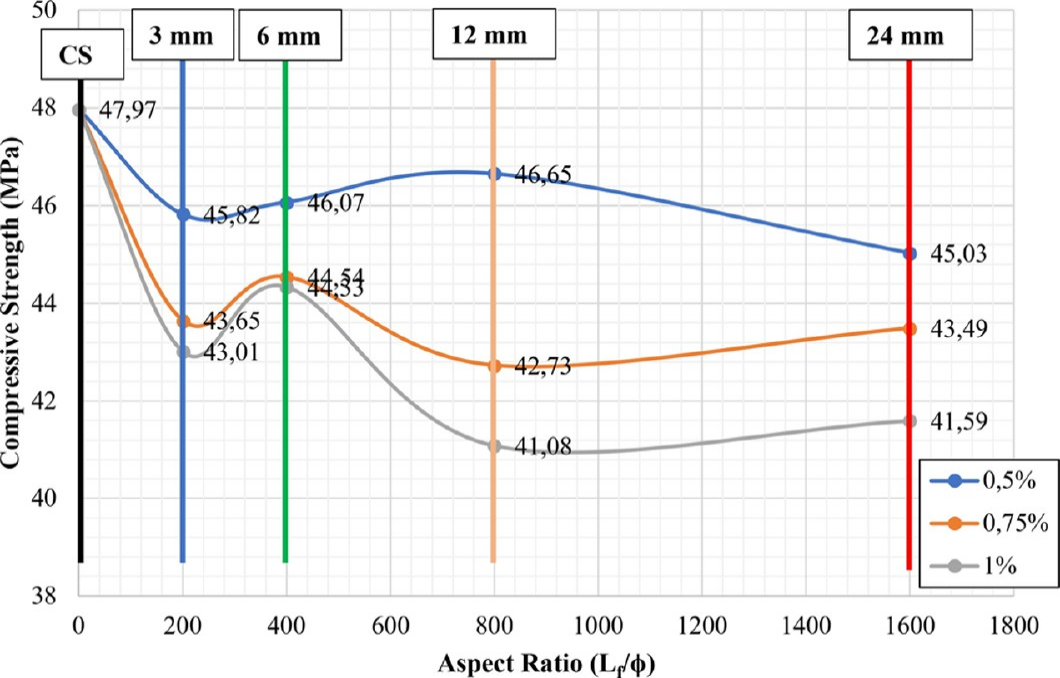
Relationship between compressive strength and aspect ratios for non-hybrid samples.

**Figure 10:**
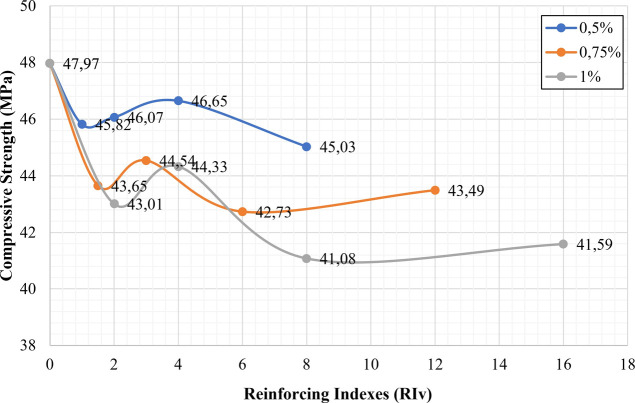
Relationship between compressive strength and reinforcing indexes for non-hybrid samples.

**Figure 11:**
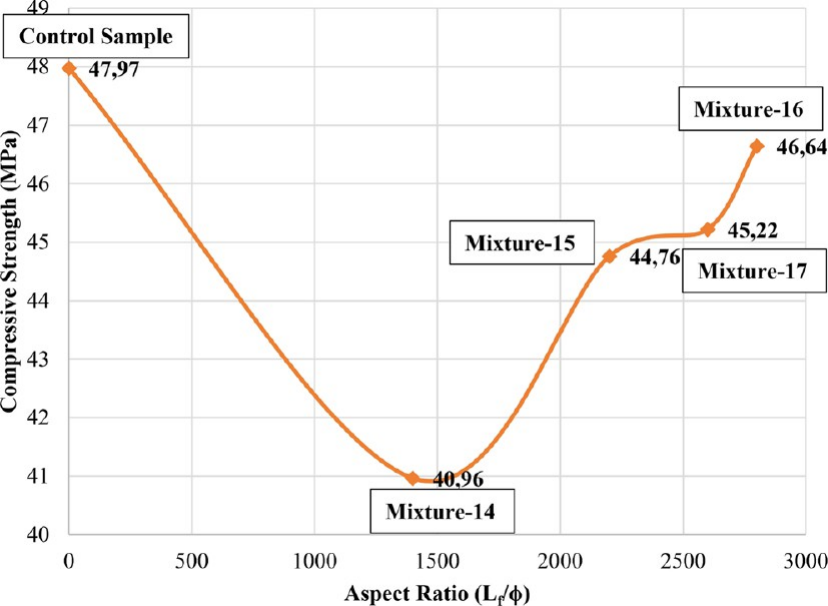
Relationship between compressive strength and aspect ratios for hybrid samples.

**Figure 12:**
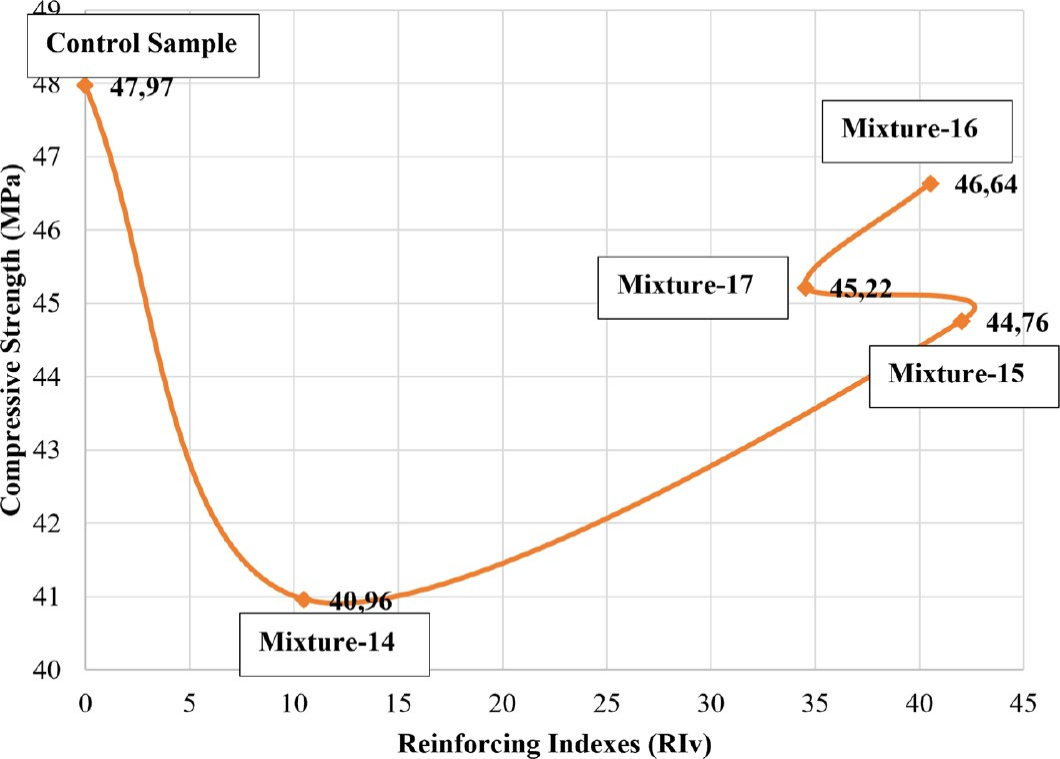
Relationship between compressive strength and reinforcing indexes for hybrid samples.

When Figures 5, 6, and 8 were analyzed, the increasing trend observed in the flexural strengths of samples was not observed in the compressive strength. In terms of compressive strength, the most remarkable result was that all of the CGFRCs had compressive strengths that were lower than the control sample (Figures 9, 10, 11, and 12). Similarly, a decrease in the compressive strength of the samples with the fiber additive has been observed by Seker et al.^29^ Seker et al.^29^ stated in their studies that the compressive strengths decreased by 3.5 to 20% with the addition of different percentages of chopped fiber.

**Figure 13:**
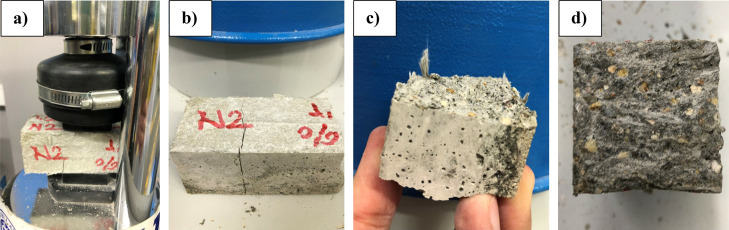
Fracture modes and chopped fiber behavior after fracture: (a,b) fracture modes, (c) fracture surface, and (d) chopped fiber on the surface.

In the experimental studies carried out within the scope of the study, the workability and the fracture mechanics of the samples were also investigated. With an increase in fiber additives, the mixing and workability of the samples decrease considerably. It is believed that this occurs because the water in the concrete is absorbed rapidly by the fibers, and there is not enough moisture to make it workable. Muley et al.^32^ reported that the workability of concrete significantly decreased when the fiber dosage rate increased. A similar situation was emphasized in the study conducted by Qureshi and Ahmed.^33^ The study concluded that the workability of concrete decreases significantly with the increase in fiber content. When the samples were examined in terms of fracture mechanics, it was concluded that the fiber additive also affected the fracture behavior of the samples. Concrete samples typically exhibited brittle behavior, while fiber-reinforced concrete exhibited ductility. As a result of the experiments, it has been observed that all of the fractured samples were ductile. With an increase in fiber count and fiber size, it has been observed that the samples did not spontaneously split as a result of the flexural strength test. Furthermore, no sudden breakage or scattering was observed in any of the tests for compressive strength on any of the samples. Figure 13 illustrates the fracture modes. As can be seen from the figure, no rupture of the fibers was observed due to the experiment. According to the investigation, it has been determined that fibers vertical to the crushed surface are pulled out from the concrete and remain on the surface. There may be a hypothesis that the high tensile strength of glass fibers prevents the samples from shattering under the applied load, which also prevents the brittle fracture of the samples. Similar investigations were reported by Cakir^14^ and Seker et al.^29^ The previous studies stated that the chopped fibers affect the failure modes and the load transfer pattern.

**Figure 14:**
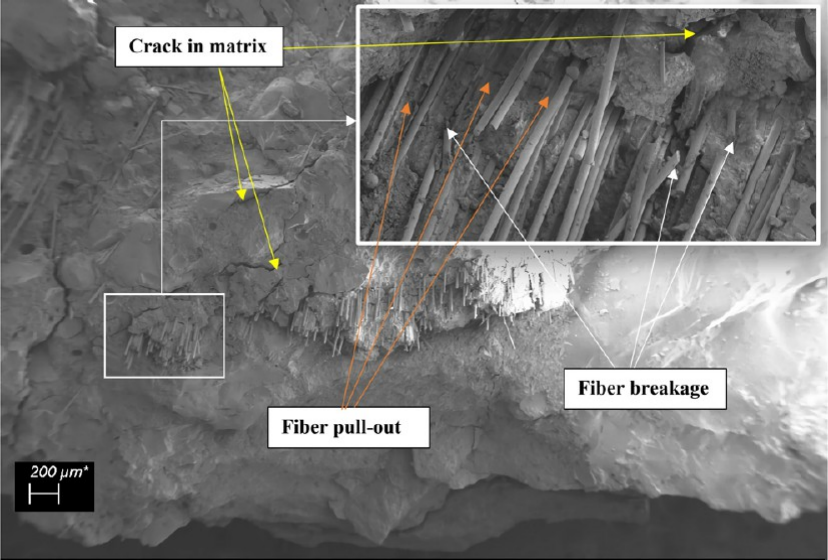
Fiber pull-out, fiber breakage, and crack in matrix examples in mixture-13.

**Figure 15:**
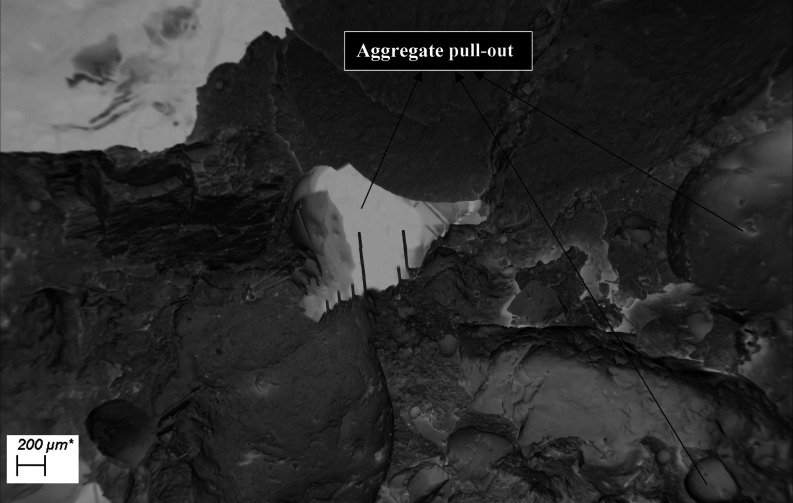
Aggregate pull-out examples in mixture-2.

**Figure 16:**
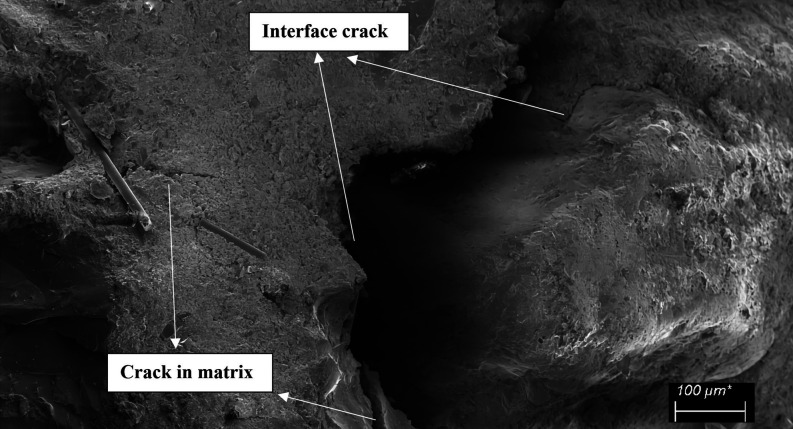
Interface cracks in mixture-14.

When the damaged CGFRC samples were evaluated by using DM and SEM images, the CGFs were found to attract and prevent the matrix from spreading between the aggregates. The most common problems were interface cracks, fiber breakages, fiber and aggregate pull-outs, and fiber agglomerations resulting in air voids (Figures 14, 15, and 16). The matrix and interfaces were also cracked from fibers and aggregates that were dislodged or broken. This caused the aggregates to loosen and dislocate without being damaged. As a result of the excessive agglomeration of fibers with a fiber ratio of 1.0%, concrete was not permeated between some fibers and huge air voids developed between them. As a result of the loosening of the aggregate, damage was generally observed. A weak aggregate–matrix interface indicates that the aggregate strength was weaker than the matrix strength, which is normally expected to be strong. As the fiber ratio increases, these problems become more intense and negative.

**Figure 17:**
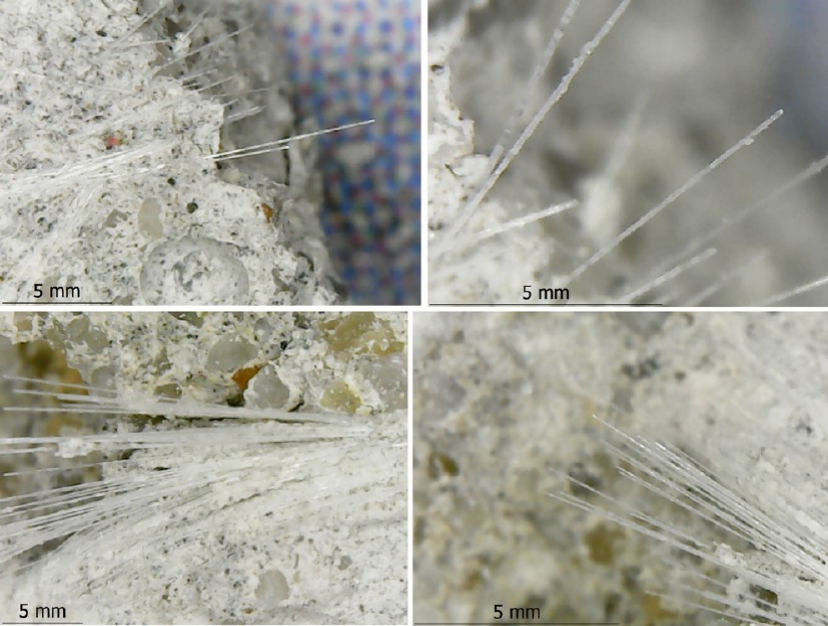
Separation of fibers into filaments.

**Figure 18:**
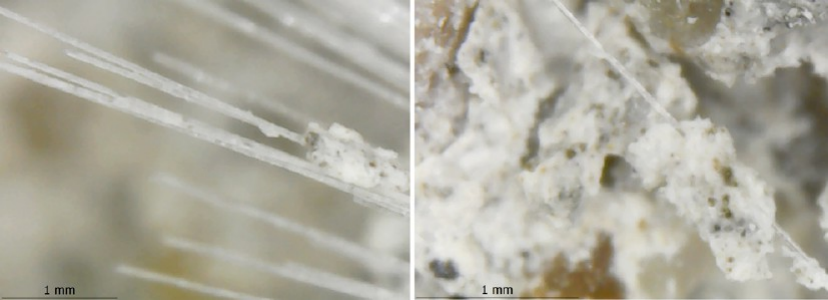
Subsided concrete on some of the fiber filaments.

**Figure 19:**
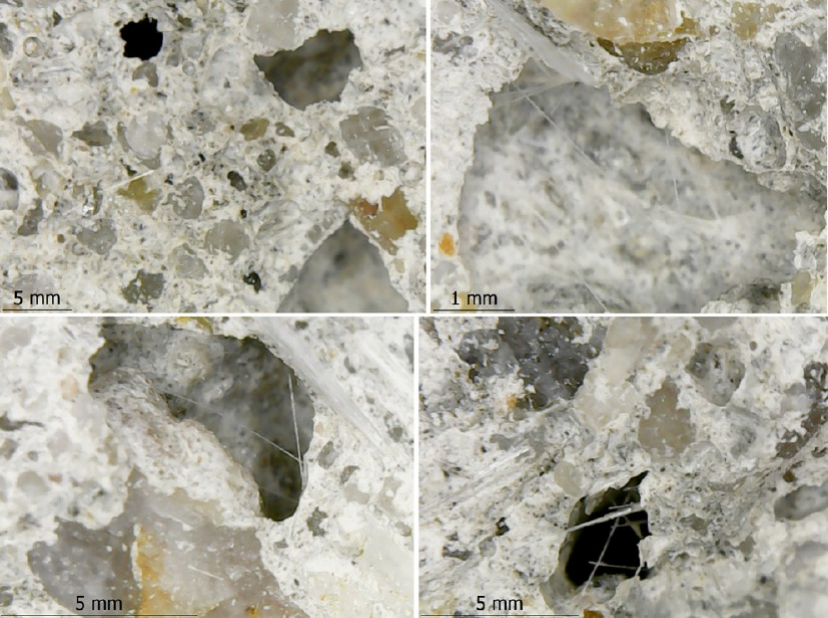
Dislodged aggregate holes.

**Figure 20:**
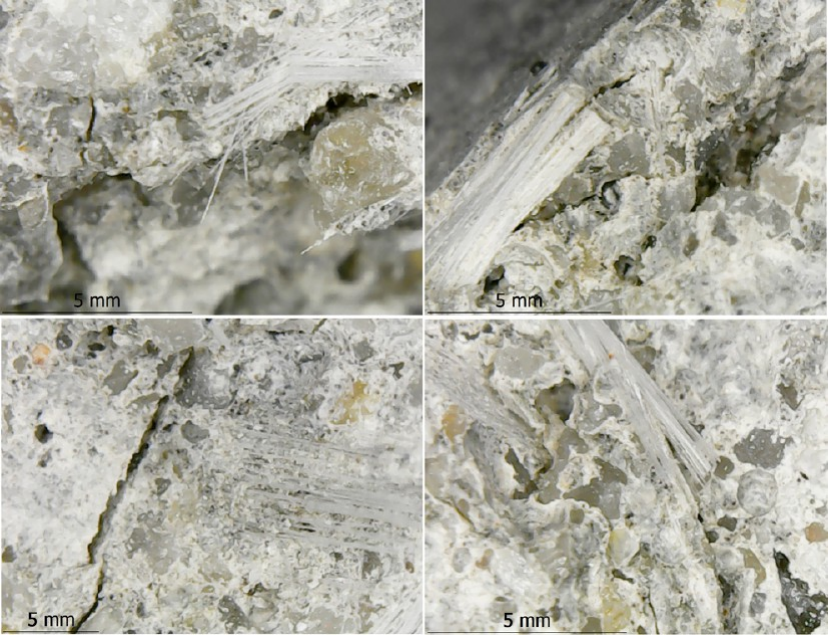
Interface and matrix cracks.

While mixing the components, the chopped fibers were separated into individual fiber filaments. By separating fibers into individual filaments (Figure 17), fibers and concrete were bonded better as the interfaces were increased. As seen from the DM images, the concrete subsided locally on some of the fiber filaments (Figure 18) and did not spread entirely on the filament surfaces. It was believed that these conditions contribute to interfacial cracking between fibers and matrixes. Moreover, the DM images also showed resolved aggregate holes (Figure 19). Some aggregates might have become dislodged from the matrix due to the smoother surfaces of the aggregates. In some cases, the holes could be indicated that the aggregate and matrix interfaces were completely detached, and it was indicated that the aggregate and matrix interfaces were weaker than the aggregate itself (Figure 20).

## 6 Conclusions and Suggestions

Although concrete is a strong material in terms of compression, it is brittle and has a low tensile strength. The most preferred method of improving the mechanical properties of concrete is adding additives or reinforcing concrete with different materials. With the advances in composite technology, the reinforcement of concrete with chopped fibers has become among the major reinforcement materials for concrete. This study examines the effects of high aspect ratios and reinforcing indexes on the mechanical properties of hybrid and non-hybrid CGFRC. In this study, the GCFs (fiber diameter, ϕ = 0.015 mm) with four different volume fractions (0, 0.5, 0.75, and 1%) and four different lengths (3, 6, 12, and 24 mm) were mixed into the concrete considering the aspect ratios between 200 and 2800, and the reinforcing indexes between 1 and 42. A total of 51 samples were prepared for the study that included 3 control, 36 non-hybrid, and 12 hybrid samples. Then, the flexural strength and compressive strength tests were conducted on the CGFRC samples.

Experimental studies have demonstrated that fiber reinforcement has both positive and negative effects on the mechanical properties of concrete. Specifically, CGF reinforcement has a positive effect on the flexural strength of concrete, but a negative effect on its compressive strength. In hybrid samples, the flexural strength increased with higher aspect ratios and reinforcing index values, whereas non-hybrid samples did not experience this increase. In terms of compressive strength, all CGFRC samples have lower compressive strengths compared to the control sample. Additionally, it is determined that the mixing and workability of the samples considerably decrease with an increase in the fiber volume fraction, and the increase of the fiber volume fraction causes brittle fractures in concretes to be transformed into ductile fractures. In most samples, interface cracks, fiber breakage, fiber and aggregate pull-outs, fiber agglomerations, and air voids are common problems. These problems, however, become more intense and negative as fiber ratios increase.

Although it is expected that the study will contribute significantly to the literature, it is essential to conduct similar studies on samples with low fiber ratios. Moreover, the study considers only CGF and certain fiber sizes and fiber ratios. Due to these reasons, it is recommended to conduct additional studies with different types of fibers, different fiber lengths, and different fiber ratios.
